# The gut microbiota-independent virulence of noninvasive bacterial pathogen *Citrobacter rodentium*

**DOI:** 10.1371/journal.ppat.1012758

**Published:** 2024-12-04

**Authors:** Yue Liu, Dongqing Xu, Songwei Guo, Shuyu Wang, Hua Ding, Catherine Siu, Fengyi Wan

**Affiliations:** 1 Department of Biochemistry and Molecular Biology, Bloomberg School of Public Health, Johns Hopkins University, Baltimore, Maryland, United States of America; 2 Department of Molecular Microbiology and Immunology, Bloomberg School of Public Health, Johns Hopkins University, Baltimore, Maryland, United States of America; 3 Department of Oncology, Sidney Kimmel Comprehensive Cancer Center, Johns Hopkins University, Baltimore, Maryland, United States of America; University of California Davis School of Medicine, UNITED STATES OF AMERICA

## Abstract

Attaching and effacing (A/E) bacterial pathogens consist of human pathogens enteropathogenic *Escherichia coli*, enterohemorrhagic *E*. *coli* and their murine equivalent *Citrobacter rodentium* (CR). Emerging evidence suggests that the complex pathogen-microbiota-host interactions are critical in conferring A/E pathogen infection-induced severe symptoms and lethality in immunocompromised hosts; however, the precise underlying mechanisms remain enigmatic. Here we report that CR infection causes severe colitis and mortality in interleukin 22 knockout (*Il22*^*-/-*^) and Rag1 knockout (*Rag1*^*-/-*^) mice under germ-free (GF) conditions. In a gut microbiota-independent manner, CR colonizes in GF *Il22*^*-/-*^ and *Rag1*^*-/-*^ animals, triggers colonic epithelial tissue damage and systemic dissemination of CR, and results in lethal infections. Pretreatment with cefoxitin, a broad-spectrum antibiotic, exacerbates CR-induced colitis and lethality in specific-pathogen-free (SPF) *Il22*^*-/-*^ and *Rag1*^*-/-*^ mice. Together our results reveal that CR possesses a gut microbiota-independent virulence, which is better illustrated during infections in immunocompromised hosts associated with severe outcomes.

## Author summary

We reveal that *Citrobacter rodentium* (CR) colonizes in the cecum and the colon and causes severe symptoms and lethality in germ-free (GF) *Il22*^*-/-*^ and *Rag1*^*-/-*^ mice, independent of gut microbiota. Pretreatment of antibiotics in specific-pathogen-free (SPF) immunocompromised animals exacerbates CR-induced lethality. These results suggest that CR possesses gut microbiota-independent capacity in colonization and virulence and the commensal bacteria act as the front-line defense against CR infection.

## Introduction

Human pathogens enteropathogenic *Escherichia coli* (EPEC) and enterohemorrhagic *E*. *coli* (EHEC) are among the leading etiological agents for foodborne diseases [1,2]. Murine pathogen *Citrobacter rodentium* (CR) shares most pathogenic mechanisms and virulence factors with EPEC and EHEC [3–8]; hence CR infection in mice has been widely used as a small animal model to study the underlying pathogenic mechanisms. Upon infection in specific-pathogen-free (SPF) mice, CR adapts to the gastrointestinal tract with expression of an array of virulence genes [9]. After reaching peak colonization, CR burden gradually declines, with complete clearance observed 2–4 weeks post infection [9]. While gut microbiota is generally regarded as a barrier against enteric pathogen colonization in SPF mice, certain Kanamycin-sensitive commensal species appear essential for CR to maintain colonic tissue association during peak of infection [10]. Of note, wild-type C57Bl/6 and many immunocompetent strains show low or no mortality, whereas immunocompromised animals, for instance interleukin-22 knockout (*Il22*^*-/-*^) and interleukin-22 receptor subunit alpha 1 knockout (*Il22ra1*^*-/-*^) mice, exhibit markedly elevated mortality following CR challenge [11,12]. Systemic spread of CR was reported in the infected SPF *Il22*^*-/-*^ mice [11–13]; in contrast, Pham and colleagues found that systemic dissemination of *Enterococcus faecalis*, an intestinal opportunistic pathogen, led to lethality in *Il22ra1*^*-/-*^ mice [11]. It has remained elusive the severe symptoms and outcomes in CR-infected immunocompromised SPF animals attribute to the systemic dissemination of CR *per se*, certain commensal microbes, or the synergic pathogen-microbiota interactions.

Here, utilizing germ-free (GF) *Il22*^*-/-*^ mice lacking the critical cytokine Il22 for an acute phase response and Rag1 knockout (*Rag1*^*-/-*^) mice lacking mature T and B lymphocytes in adaptive immune response, both of which are susceptible to CR infection under SPF condition, we assessed the impact of gut microbiota on the CR susceptibility. Our findings reveal that in the absence of gut microbiota, CR infection sufficiently colonizes on the colonic epithelium and triggers tissue damage, thus causing systemic dissemination of CR and resulting in animal lethality. Such severe symptoms strongly indicate that CR possesses virulence independent of the host gut microbiota, which is better illustrated in the infected immunocompromised GF *Il22*^*-/-*^ and *Rag1*^*-/-*^ animals.

## Results

### CR infection causes lethality in both SPF and GF *Il22*^-/-^ mice

**Fig 1:**
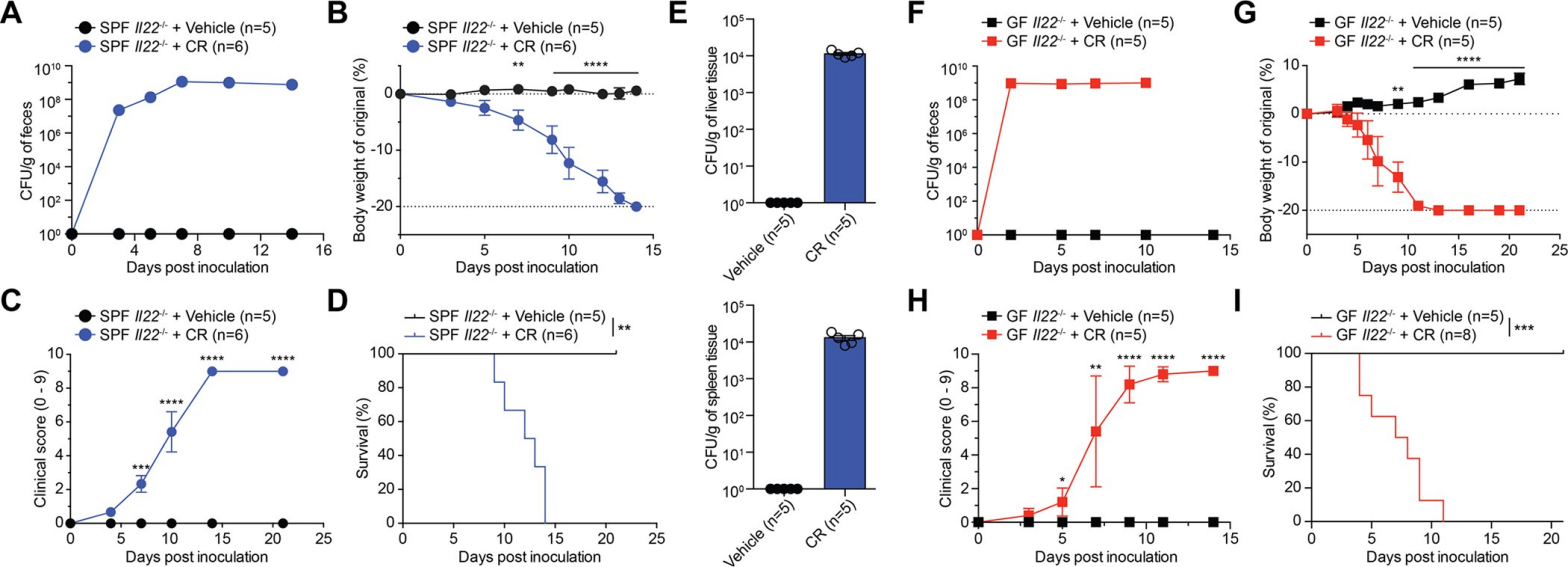
CR causes lethal infection in SPF and GF *Il22*^-/-^ mice. (**A-D**) Fecal live *Citrobacter rodentium* (CR) burden (A), body weight loss (B), clinical scores (C), and survival (D) of specific-pathogen-free (SPF) *Il22*^*-/-*^ mice in C57Bl/6J background at indicated days post inoculation (dpi) with 2 × 10^9^ CFU of CR or vehicle control. (**E**) CR burdens in the liver (*top*) and the spleen (*bottom*) derived from SPF *Il22*^*-/-*^ mice at 10 dpi infected with vehicle control or CR. No CR burden was detected in vehicle controls. (**F-I**) Fecal live CR burden (F), body weight loss (G), clinical scores (H) and survival (I) of germ-free (GF) *Il22*^*-/-*^ mice at indicated dpi with 2 × 10^9^ CFU of CR or vehicle control. Data are representative results of at least two independent experiments. The error bars (A and F) are too small to be visible. ** *p* < 0.01, *** *p* < 0.001, **** *p* < 0.0001 with Student’s *t* tests (A-C, and G-H) and Long-rank test (D and I).

CR infection leads to severe symptoms and lethality in an array of immunocompromised mouse strains [14]. Consistent to previously reported [11–13,15], CR infection in SPF *Il22*^-/*-*^ mice resulted in severe body weight loss, watery diarrhea, and 100% mortality by 14 days post inoculation (dpi) (Fig 1A–1D). Moreover, systemic spread of CR associated with compromised colonic epithelial barrier integrity occurred in CR-infected mice, in contrast to no colony-forming units (CFU) of CR detected in the controls (Fig 1E). While these results highlight that CR could cause sepsis-associated lethality under SPF condition, it remains puzzling whether the lethality in SPF *Il22*^-/-^ animals attributes to the spread of CR *per se* or the synergistic CR-gut microbiota interactions.

We rederived germ-free (GF) *Il22*^-/-^ mice and orally infected them with CR or vehicle control, to assess the possible virulence of CR in the absence of commensal microbiota. In contrast to the gradual increase in CR burdens in SPF animals (Fig 1A), CR colonized maximally in GF *Il22*^*-/-*^ mice as early as 2 dpi (Fig 1F). The CR-infected GF *Il22*^-/-^ mice, starting from 4 dpi, exhibited severe clinical symptoms and 100% lethality by 11 dpi (Fig 1G–1I). These results demonstrate that regardless of gut microbiota, CR alone is sufficient to cause symptoms and lethality in GF *Il22*^-/-^ mice, indicating the gut microbiota-independent virulence of CR.

### CR colonization causes lethal colitis in GF *Il22*^-/-^ mice

**Fig 2:**
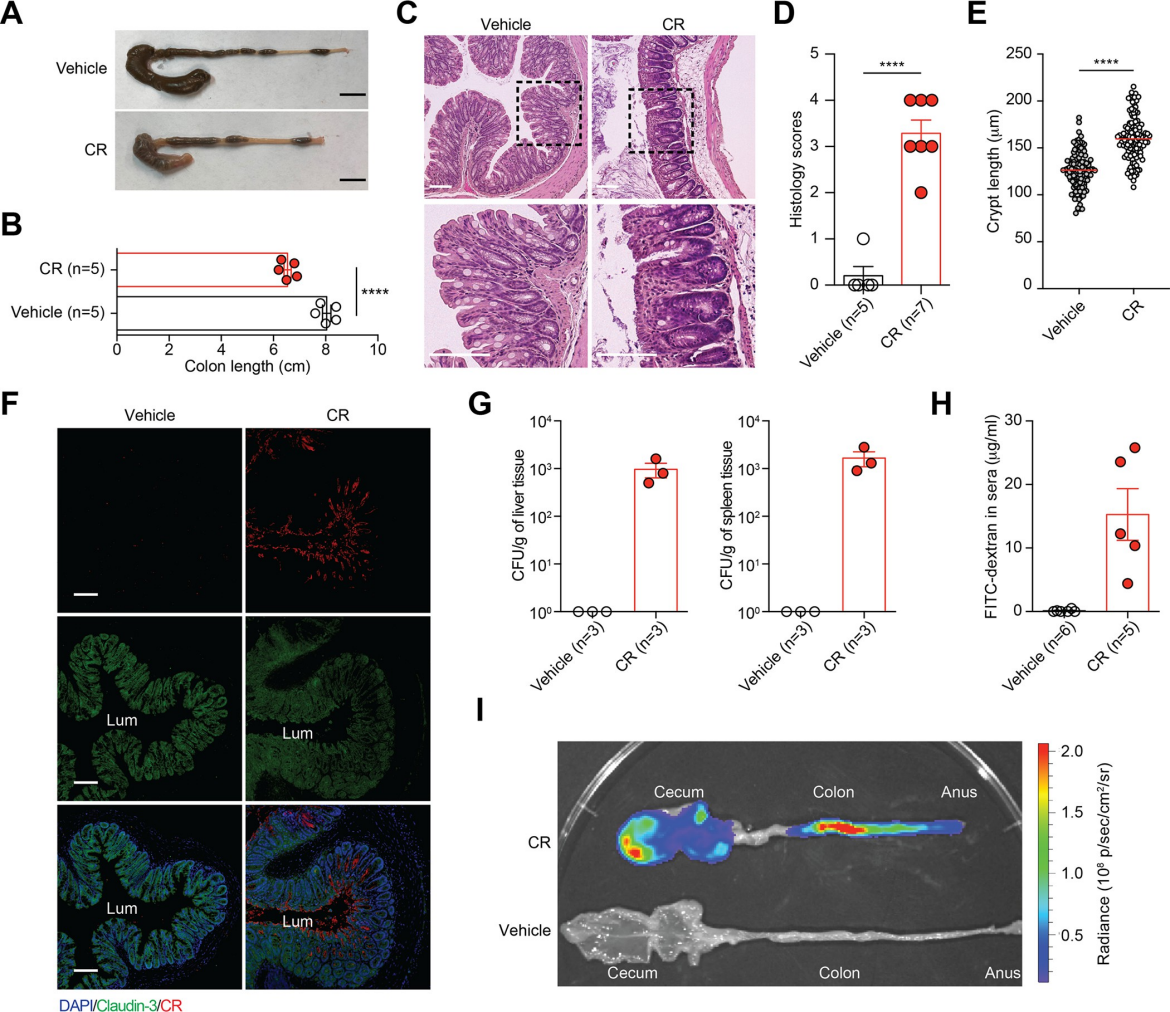
CR colonization causes lethality in GF *Il22*^-/-^ mice. (**A**) Representative images of the cecum and the colon derived from GF *Il22*^*-/-*^ mice at 5 days post inoculation (dpi) infected with vehicle control or 2 × 10^9^ CFU of CR. Scale bars, 1 cm. (**B**) Colon lengths of GF *Il22*^*-/-*^ mice infected with vehicle control or CR. (**C-D**) Hematoxylin and eosin staining (C) and histopathology scores (D) of colon sections derived from GF *Il22*^*-/-*^ mice at 5 dpi infected with vehicle control or CR. Scale bars, 100 μm. (**E**) Crypt lengths measured in colonic tissue sections from at least 4 GF *Il22*^*-/-*^ mice infected with CR or vehicle control at 5 dpi. (**F**) Immunofluorescence micrographs of Claudin-3 and CR in the colons derived from GF *Il22*^*-/-*^ mice at 5 dpi infected with vehicle control or CR, with nuclei counterstained by DAPI. Lum indicates the colon luminal space. Scale bars, 100 μm. (**G**) Live CR burdens in the liver (*left*) and the spleen (*right*) derived from GF *Il22*^*-/-*^ mice at 5 dpi infected with vehicle control or CR. No CR burden was detected in vehicle controls. (**H**) FITC-dextran concentrations in the sera of GF *Il22*^*-/-*^ mice at 5 dpi infected with vehicle control or CR, at 4 h post oral administration of FITC-dextran. (**I**) Representative *ex vivo* tissue images of GF *Il22*^*-/-*^ mice infected with vehicle control or CR at 5 dpi, with an IVIS50 camera and displayed as pseudo colors. The color scale bar indicates bioluminescence signal intensity (photons sec^-1^ cm^-2^ sr^-1^). Data are representative results of at least two independent experiments. **** *p* < 0.0001 with Student’s *t* tests (B, D and E).

At necropsy, the colon lengths of CR-infected GF *Il22*^-/-^ mice were substantially shortened compared to the controls (Fig 2A and 2B). Moreover, histopathological analyses on the colons revealed that CR infection led to more profound epithelial damage, goblet cell depletion, immune cell infiltration, and the hallmark colonic crypt hyperplasia [16] in the infected GF mice (Fig 2C–2E), which indicates that CR alone resulted in profound colonic inflammation in the GF *Il22*^-/-^ animals. Moreover, in the infected colons, CR induced significant redistribution and degradation of Claudin-3, one of the most abundant tight junction proteins [17] (Fig 2F). Consistently, CR burdens were augmented in the liver and the spleen of CR-infected animals but remained not detectable in the controls (Fig 2G). Indeed, CR-caused damage to the integrity of colonic epithelial barrier was supported by the increased gut permeability in the infected GF *Il22*^-/-^ mice, as measured by the leakage of gut-derived FITC-dextran to the serum (Fig 2H). Furthermore, utilizing *ex vivo* bioluminescent imaging of ICC180 strain, we demonstrated that CR attached to the cecum and the colon of infected GF *Il22*^-/-^ mice at 5 dpi (Fig 2I). These results suggest that CR infection causes *bona fide* colonization with tight attachment to the cecal and colonic epithelia, thus triggering tight junction disruption, and leading to systemic dissemination of CR and mortality in GF *Il22*^-/-^ mice.

### CR causes lethal infection in GF *Rag1*^-/-^ mice

**Fig 3:**
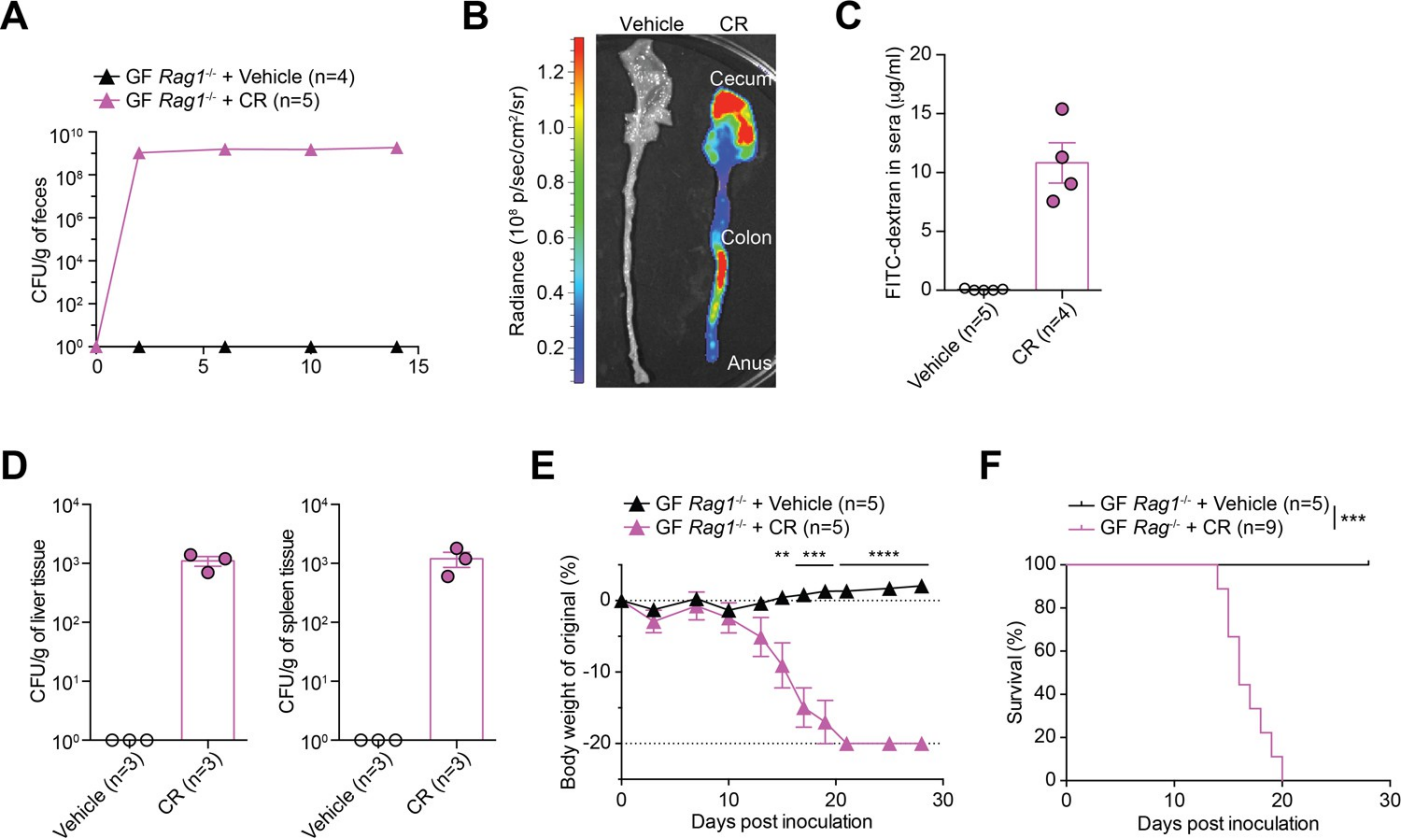
CR colonization leads to lethal colitis in GF *Rag1*^-/-^ mice. (**A**) Fecal live CR burden of germ-free (GF) *Rag1*^-/-^ mice at indicated dpi with 2 × 10^9^ CFU of CR or vehicle control. (**B**) Representative *ex vivo* tissue images of GF *Rag1*^-/-^ mice infected with vehicle control or CR at 5 days post inoculation (dpi), with an IVIS50 camera and displayed as pseudo colors. The color scale bar indicates bioluminescence signal intensity (photons sec^-1^ cm^-2^ sr^-1^). (**C**) FITC-dextran concentrations in the sera of GF *Rag1*^-/-^ mice at 14 dpi infected with vehicle control or CR, at 4 h post oral administration of FITC-dextran. (**D**) Live CR burdens in the liver (*left*) and the spleen (*right*) derived from GF *Rag1*^-/-^ mice at 14 dpi infected with vehicle control or CR. No CR burden was detected in vehicle controls. (**E-F**) Body weight loss (E) and survival (F) of GF *Rag1*^-/-^ mice at indicated dpi with vehicle control or CR. Data are representative results of at least two independent experiments. ** *p* < 0.01, *** *p* < 0.001, **** *p* < 0.0001, with Student’s *t* tests (E) and Long-rank test (F).

To examine whether the lethal infection of CR occurs restrictedly in *Il22*^-/-^ mice, we carried out oral CR infection in GF *Rag1*^-/-^ mice, another immunocompromised strain where the V(D)J recombination activation gene *Rag1* was deleted [18]. Indeed, CR colonized maximally in GF *Rag1*^-/-^ mice as early as 2 dpi (Fig 3A). The *ex vivo* bioluminescent imaging ascertained the tight attachment of CR to the cecum and the colon of infected GF *Rag1*^-/-^ animals at 5 dpi (Fig 3B). Of note, such colonization sufficiently resulted in the damage to colonic epithelial barrier and CR systemic dissemination, as evidenced by the augmented serum levels of FITC-dextran leaked from the gut (Fig 3C) and the elevated CR burdens in the liver and the spleen of CR-infected GF *Rag1*^-/-^ mice at 14 dpi, compared with the controls (Fig 3D). Indeed, GF *Rag1*^-/-^ mice suffered severe body weight loss (Fig 3E) and succumbed to CR infection by 16–20 dpi (Fig 3F). Under GF condition, infection of CR in both *Rag1*^-/-^ and *Il22*^-/-^ mice is sufficient to cause severe symptoms and mortality, supporting that CR indeed harbors gut microbiota-independent virulence.

### Gut microbiota protects CR infection-caused lethality in SPF animals

**Fig 4:**
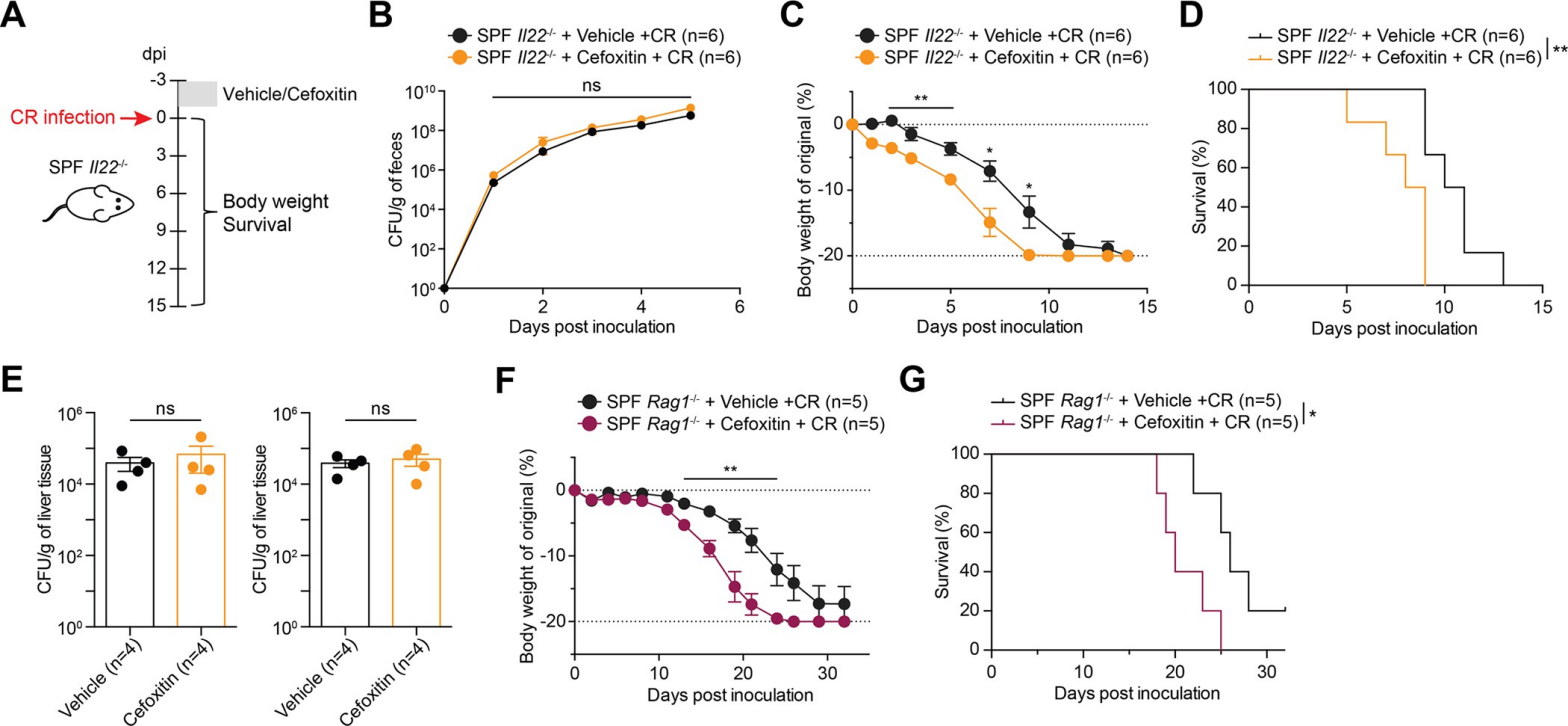
The gut microbiota offers protection against CR-caused lethality in SPF *Il22*^-/-^ and *Rag1*^-/-^ mice. (**A**) Schematic of *Citrobacter rodentium* (CR) infection experiment in specific-pathogen-free (SPF) *Il22*^*-/-*^ mice. The indicated mice were pretreated with normal drinking water (Vehicle) or drinking water containing Cefoxitin (500 mg/L) for 48 h. 24 h after removal of antibiotic-containing drinking water, the mice were orally inoculated with 2 × 10^9^ CFU of CR. (**B-D**) Fecal live CR burden (B), body weight loss (C) and survival (D) of vehicle control- or Cefoxitin-pretreated SPF *Il22*^*-/-*^ mice at indicated days post inoculation (dpi) with CR. (**E**) CR burdens in the liver (*left*) and the spleen (*right*) derived from vehicle control- or Cefoxitin-pretreated SPF *Il22*^*-/-*^ mice at 5 dpi infected with CR. (**F-G**) SPF *Rag1*^-/-^ mice were pretreated with normal (Vehicle) or Cefoxitin-containing drinking water, followed with CR infection, as described in (A). Body weight loss (F) and survival (G) of vehicle control- or Cefoxitin-pretreated SPF *Rag1*^-/-^ mice at indicated dpi with 2 × 10^9^ CFU of CR. Data are representative results of at least two independent experiments. ns, not significant; * *p* < 0.05, ** *p* < 0.01, with Student’s *t* tests (C and F) and Long-rank test (D and G).

We observed that CR-caused mortality in SPF *Il22*^-/-^ mice occurred noticeably slower compared with that in GF *Il22*^-/-^ animals (Fig 1D and 1I), indicating that gut microbiota could protect the host against CR infection. Hence, we modified SPF *Il22*^-/-^ mouse gut microbiota via pretreatment with Cefoxitin, a broad-spectrum antibiotic, and assessed its impact on the mouse susceptibility to CR challenge (Fig 4A). Fecal CR burdens were marginally augmented in Cefoxitin-administrated SPF *Il22*^-/-^ mice compared to the vehicle-treated animals (Fig 4B). Notably, Cefoxitin-pretreated animals became more susceptible to CR infection, with accelerated body weight loss and mortality (Fig 4C and 4D), albeit comparable CR burdens observed in the liver and the spleen (Fig 4E). Similarly, SPF *Rag1*^-/-^ mice became more vulnerable to CR infection following Cefoxitin administration, as evidenced by augmented body weight loss and mortality (Fig 4F–4G). These results indicate that in SPF *Il22*^-/-^ and *Rag1*^-/-^ mice, gut microbiota act as a protective barrier against CR infection.

## Discussion

CR is known to cause self-limiting infection in a wide range of immunocompetent mouse strains including C57Bl/6 mice [9]. While substantial changes in host gut microbiota composition were reported during CR infections [19,20], the impact of gut microbiota on CR colonization and virulence has been studied with controversial findings [10,21–24]. Utilizing SPF and GF C57Bl/6 mice together with CR reporter strains, Mullineaux-Sanders and colleagues proposed that while expression of virulence genes is not sufficient for CR colonization at the colonic mucosa, certain Kanamycin-sensitive species in gut microbiota seem essential for CR colonization during the peak of infection [10]. However, CR was previously shown attaching to the cecal and colonic epithelia with characterized attaching/effacing lesions in GF C57Bl/6 mice [23], indicating the gut microbiota-independent colonization of CR. Moreover, Campbell et al. reported that gut microbiota creates the dominant bottleneck in preventing CR infection [22]. The protective effect of gut microbiota on CR colonization and virulence was also supported by the evidence that antibiotic treatment predisposes the SPF C57Bl/6 mice to exacerbated CR-induced colitis [24]. Of note, the majority of these findings was performed using immunocompetent animals, which only exhibit transient and mild symptoms following CR infection. In contrast, in the immunocompromised GF *Il22*^*-/-*^ mice, we showed that CR infection leads to 100% mortality, coinciding with severe colonic barrier damage and systemic dissemination of CR. These results demonstrate that CR *per se* is sufficient for the pathological infection course in GF *Il22*^*-/-*^ mice. The tight attachment of CR to the cecum and the colon, independent of gut microbiota, most likely represents a *bona fide* colonization, which is further supported by the associated severe symptoms and outcomes. Moreover, CR elicits lethal infection in GF *Rag1*^-/-^ mice that harbor distinct host deficiencies from GF *Il22*^*-/-*^ animals, further strengthening the notion that CR harbors gut microbiota-independent virulence. Of note, accumulating evidence underscores that A/E pathogen infections lead to more severe symptoms and lethality in immunocompromised populations [25, 26]. Our findings illustrate a previously underappreciated gut microbiota-independent virulence of CR, which offers new insights into the complex pathogenic mechanisms during A/E pathogen infections.

## Materials and Methods

### Ethics statement

All animal experiments were performed according to the protocol approved by Animal Care and Use Committee at Johns Hopkins University and in direct accordance with the National Institutes of Health (NIH) guidelines. *Il22*^-/-^ and *Rag1*^-/-^ mice in C57Bl/6J background were maintained in a specific-pathogen-free (SPF) or germ-free (GF) mouse facility, as previously described [27].

### Bacterial strains and growth conditions

Wild-type CR (DBS 100 strain) and bioluminescent CR (ICC180 strain) were described previously [28]. CR strains were grown in LB broth at 37°C overnight with shaking from single colonies on Luria–Bertani (LB) plates.

### Bacterial infection in mice

CR infection in SPF and GF mice was conducted as we did previously [29, 30]. For antibiotics treatment of SPF animals, mice were given water containing 500 mg/L Cefoxitin for 48 h; after removal of antibiotic water for 24 h, mice were orally inoculated with CR.

### Histology, immunohistochemistry, and immunofluorescence on colon tissue sections

GF *Il22*^-/-^ and *Rag1*^-/-^ mice were euthanized at 5 dpi and 14 dpi, respectively, as they succumbed to CR infection starting around 5 dpi and 14 dpi, respectively. Histology, immunohistochemistry, and immunofluorescence staining of colon tissue sections were performed as previously described [31]. Colonic crypt hyperplasia was measured as previously reported [16].

### *Ex vivo* bioluminescent imaging

The *ex vivo* bioluminescent imaging of CR burden was conducted as previously described [28].

### FITC-dextran assays

FITC-dextran assays for colonic permeability were performed as previously described [32].

### Statistical analysis

Statistical analysis was performed using GraphPad Prism version 9.0.1 (GraphPad Software, San Diego, CA). Standard errors of means (s.e.m.) were plotted in graphs. Significant differences were detailed in the figure legends.

## Supporting information

S1 Data The underlying numerical data for the figure panels. An Excel spreadsheet containing, in separate sheets, the underlying numerical data for Figs 1A, 1B, 1C, 1D, 1E, 1F, 1G, 1H, 1I, 2B, 2D, 2E, 2G, 2H, 3A, 3C, 3D, 3E, 3F, 4B, 4C, 4D, 4E, 4F and 4G.
(XLSX)

## References

[1] Hu J, Torres AG. Enteropathogenic Escherichia coli: foe or innocent bystander? Clin Microbiol Infect. 2015;21(8):729–34. Epub 20150128. doi: 10.1016/j.cmi.2015.01.015 ; PubMed Central PMCID: PMC4497942. 25726041 PMC4497942

[2] Ochoa TJ, Contreras CA. Enteropathogenic escherichia coli infection in children. Curr Opin Infect Dis. 2011;24(5):478–83. doi: 10.1097/QCO.0b013e32834a8b8b ; PubMed Central PMCID: PMC3277943. 21857511 PMC3277943

[3] Garmendia J, Frankel G, Crepin VF. Enteropathogenic and enterohemorrhagic Escherichia coli infections: translocation, translocation, translocation. Infect Immun. 2005;73(5):2573–85. Epub 2005/04/23. doi: 10.1128/IAI.73.5.2573-2585.2005 ; PubMed Central PMCID: PMC1087358. 15845459 PMC1087358

[4] Nougayrede JP, Fernandes PJ, Donnenberg MS. Adhesion of enteropathogenic Escherichia coli to host cells. Cell Microbiol. 2003;5(6):359–72. Epub 2003/06/05. doi: 10.1046/j.1462-5822.2003.00281.x . 12780774

[5] Santos AS, Finlay BB. Bringing down the host: enteropathogenic and enterohaemorrhagic Escherichia coli effector-mediated subversion of host innate immune pathways. Cell Microbiol. 2015;17(3):318–32. Epub 2015/01/16. doi: 10.1111/cmi.12412 . 25588886

[6] Allaire JM, Crowley SM, Law HT, Chang SY, Ko HJ, Vallance BA. The Intestinal Epithelium: Central Coordinator of Mucosal Immunity. Trends Immunol. 2018;39(9):677–96. Epub 2018/05/03. doi: 10.1016/j.it.2018.04.002 . 29716793

[7] Torres AG, Zhou X, Kaper JB. Adherence of diarrheagenic Escherichia coli strains to epithelial cells. Infect Immun. 2005;73(1):18–29. Epub 2004/12/25. doi: 10.1128/IAI.73.1.18-29.2005 ; PubMed Central PMCID: PMC538947. 15618137 PMC538947

[8] Wong AR, Pearson JS, Bright MD, Munera D, Robinson KS, Lee SF, et al. Enteropathogenic and enterohaemorrhagic Escherichia coli: even more subversive elements. Mol Microbiol. 2011;80(6):1420–38. Epub 2011/04/15. doi: 10.1111/j.1365-2958.2011.07661.x . 21488979

[9] Wiles S, Clare S, Harker J, Huett A, Young D, Dougan G, et al. Organ specificity, colonization and clearance dynamics in vivo following oral challenges with the murine pathogen Citrobacter rodentium. Cell Microbiol. 2004;6(10):963–72. Epub 2004/09/02. doi: 10.1111/j.1462-5822.2004.00414.x . 15339271

[10] Mullineaux-Sanders C, Collins JW, Ruano-Gallego D, Levy M, Pevsner-Fischer M, Glegola-Madejska IT, et al. Citrobacter rodentium Relies on Commensals for Colonization of the Colonic Mucosa. Cell Rep. 2017;21(12):3381–9. Epub 2017/12/21. doi: 10.1016/j.celrep.2017.11.086 ; PubMed Central PMCID: PMC5746604. 29262319 PMC5746604

[11] Pham TA, Clare S, Goulding D, Arasteh JM, Stares MD, Browne HP, et al. Epithelial IL-22RA1-mediated fucosylation promotes intestinal colonization resistance to an opportunistic pathogen. Cell Host Microbe. 2014;16(4):504–16. Epub 2014/09/30. doi: 10.1016/j.chom.2014.08.017 ; PubMed Central PMCID: PMC4190086. 25263220 PMC4190086

[12] Zheng Y, Valdez PA, Danilenko DM, Hu Y, Sa SM, Gong Q, et al. Interleukin-22 mediates early host defense against attaching and effacing bacterial pathogens. Nat Med. 2008;14(3):282–9. Epub 2008/02/12. doi: 10.1038/nm1720 . 18264109

[13] Baumler AJ, Sperandio V. Interactions between the microbiota and pathogenic bacteria in the gut. Nature. 2016;535(7610):85–93. Epub 2016/07/08. doi: 10.1038/nature18849 ; PubMed Central PMCID: PMC5114849. 27383983 PMC5114849

[14] Mullineaux-Sanders C, Sanchez-Garrido J, Hopkins EGD, Shenoy AR, Barry R, Frankel G. Citrobacter rodentium–host–microbiota interactions: immunity, bioenergetics and metabolism. Nature Reviews Microbiology. 2019;17(11):701–15. doi: 10.1038/s41579-019-0252-z 31541196

[15] Melchior K, Gerner RR, Hossain S, Nuccio SP, Moreira CG, Raffatellu M. IL-22-dependent responses and their role during Citrobacter rodentium infection. Infect Immun. 2024;92(5):e0009924. Epub 20240401. doi: 10.1128/iai.00099-24 ; PubMed Central PMCID: PMC11075456. 38557196 PMC11075456

[16] Berger CN, Crepin VF, Roumeliotis TI, Wright JC, Serafini N, Pevsner-Fischer M, et al. The Citrobacter rodentium type III secretion system effector EspO affects mucosal damage repair and antimicrobial responses. PLoS Pathog. 2018;14(10):e1007406. Epub 20181026. doi: 10.1371/journal.ppat.1007406 ; PubMed Central PMCID: PMC6221368. 30365535 PMC6221368

[17] Garcia-Hernandez V, Quiros M, Nusrat A. Intestinal epithelial claudins: expression and regulation in homeostasis and inflammation. Ann N Y Acad Sci. 2017;1397(1):66–79. Epub 2017/05/12. doi: 10.1111/nyas.13360 ; PubMed Central PMCID: PMC5545801. 28493289 PMC5545801

[18] Vallance BA, Deng W, Knodler LA, Finlay BB. Mice Lacking T and B Lymphocytes Develop Transient Colitis and Crypt Hyperplasia yet Suffer Impaired Bacterial Clearance during Citrobacter rodentium Infection. Infection and Immunity. 2002;70(4):2070–81. doi: 10.1128/IAI.70.4.2070-2081.2002 11895973 PMC127821

[19] Hoffmann C, Hill DA, Minkah N, Kirn T, Troy A, Artis D, et al. Community-wide response of the gut microbiota to enteropathogenic Citrobacter rodentium infection revealed by deep sequencing. Infect Immun. 2009;77(10):4668–78. Epub 20090727. doi: 10.1128/IAI.00493-09 ; PubMed Central PMCID: PMC2747949. 19635824 PMC2747949

[20] Belzer C, Gerber GK, Roeselers G, Delaney M, DuBois A, Liu Q, et al. Dynamics of the microbiota in response to host infection. PLoS One. 2014;9(7):e95534. Epub 20140711. doi: 10.1371/journal.pone.0095534 ; PubMed Central PMCID: PMC4094490. 25014551 PMC4094490

[21] Buschor S, Cuenca M, Uster SS, Scharen OP, Balmer ML, Terrazos MA, et al. Innate immunity restricts Citrobacter rodentium A/E pathogenesis initiation to an early window of opportunity. PLoS Pathog. 2017;13(6):e1006476. Epub 20170629. doi: 10.1371/journal.ppat.1006476 ; PubMed Central PMCID: PMC5507559. 28662171 PMC5507559

[22] Campbell IW, Hullahalli K, Turner JR, Waldor MK. Quantitative dose-response analysis untangles host bottlenecks to enteric infection. Nat Commun. 2023;14(1):456. Epub 20230128. doi: 10.1038/s41467-023-36162-3 ; PubMed Central PMCID: PMC9884216. 36709326 PMC9884216

[23] Kamada N, Kim YG, Sham HP, Vallance BA, Puente JL, Martens EC, et al. Regulated virulence controls the ability of a pathogen to compete with the gut microbiota. Science. 2012;336(6086):1325–9. Epub 20120510. doi: 10.1126/science.1222195 ; PubMed Central PMCID: PMC3439148. 22582016 PMC3439148

[24] Wlodarska M, Willing B, Keeney KM, Menendez A, Bergstrom KS, Gill N, et al. Antibiotic treatment alters the colonic mucus layer and predisposes the host to exacerbated Citrobacter rodentium-induced colitis. Infect Immun. 2011;79(4):1536–45. Epub 20110214. doi: 10.1128/IAI.01104-10 ; PubMed Central PMCID: PMC3067531. 21321077 PMC3067531

[25] Borenshtein D, McBee ME, Schauer DB. Utility of the Citrobacter rodentium infection model in laboratory mice. Curr Opin Gastroenterol. 2008;24(1):32–7. Epub 2007/11/29. doi: 10.1097/MOG.0b013e3282f2b0fb . 18043230

[26] Hartland EL, Leong JM. Enteropathogenic and enterohemorrhagic E. coli: ecology, pathogenesis, and evolution. Front Cell Infect Microbiol. 2013;3:15. Epub 2013/05/04. doi: 10.3389/fcimb.2013.00015 ; PubMed Central PMCID: PMC3639409. PMC3639409 23641365

[27] Dejea CM, Fathi P, Craig JM, Boleij A, Taddese R, Geis AL, et al. Patients with familial adenomatous polyposis harbor colonic biofilms containing tumorigenic bacteria. Science. 2018;359(6375):592–7. Epub 2018/02/09. doi: 10.1126/science.aah3648 ; PubMed Central PMCID: PMC5881113. 29420293 PMC5881113

[28] Liu Y, Fu K, Wier EM, Lei Y, Hodgson A, Xu D, et al. Bacterial Genotoxin Accelerates Transient Infection-Driven Murine Colon Tumorigenesis. Cancer Discov. 2022;12(1):236–49. Epub 2021/09/05. doi: 10.1158/2159-8290.CD-21-0912 ; PubMed Central PMCID: PMC8758537. 34479870 PMC8758537

[29] Hodgson A, Wier EM, Fu K, Sun X, Yu H, Zheng W, et al. Metalloprotease NleC suppresses host NF-kappaB/inflammatory responses by cleaving p65 and interfering with the p65/RPS3 interaction. PLoS Pathog. 2015;11(3):e1004705. Epub 2015/03/11. doi: 10.1371/journal.ppat.1004705 ; PubMed Central PMCID: PMC4355070. 25756944 PMC4355070

[30] Xia X, Liu Y, Hodgson A, Xu D, Guo W, Yu H, et al. EspF is crucial for Citrobacter rodentium-induced tight junction disruption and lethality in immunocompromised animals. PLoS Pathog. 2019;15(6):e1007898. Epub 2019/06/30. doi: 10.1371/journal.ppat.1007898 ; PubMed Central PMCID: PMC6623547. 31251784 PMC6623547

[31] Fu K, Sun X, Wier EM, Hodgson A, Liu Y, Sears CL, et al. Sam68/KHDRBS1 is critical for colon tumorigenesis by regulating genotoxic stress-induced NF-kappaB activation. Elife. 2016;5. Epub 2016/07/28. doi: 10.7554/eLife.15018 ; PubMed Central PMCID: PMC4959885. 27458801 PMC4959885

[32] Xu D, Zhou S, Liu Y, Scott AL, Yang J, Wan F. Complement in breast milk modifies offspring gut microbiota to promote infant health. Cell. 2024;187(3):750–63 e20. Epub 20240118. doi: 10.1016/j.cell.2023.12.019 ; PubMed Central PMCID: PMC10872564. 38242132 PMC10872564

